# Model of Murine Ventricular Cardiac Tissue for *In Vitro* Kinematic-Dynamic Studies of Electromagnetic and *β*-Adrenergic Stimulation

**DOI:** 10.1155/2017/4204085

**Published:** 2017-08-08

**Authors:** Lorenzo Fassina, Marisa Cornacchione, Manuela Pellegrini, Maria Evelina Mognaschi, Roberto Gimmelli, Andrea Maria Isidori, Andrea Lenzi, Giovanni Magenes, Fabio Naro

**Affiliations:** ^1^Department of Electrical, Computer and Biomedical Engineering, University of Pavia, Pavia, Italy; ^2^Centre for Health Technologies (CHT), University of Pavia, Pavia, Italy; ^3^IRCCS SDN, Naples, Italy; ^4^Department of Experimental Medicine, Sapienza University of Rome, Rome, Italy; ^5^Institute of Cell Biology and Neurobiology (IBCN), National Research Council (CNR), Rome, Italy; ^6^Department of Anatomical, Histological, Forensic and Orthopaedic Sciences, Sapienza University of Rome, Rome, Italy

## Abstract

In a model of murine ventricular cardiac tissue *in vitro*, we have studied the inotropic effects of electromagnetic stimulation (frequency, 75 Hz), isoproterenol administration (10 *μ*M), and their combination. In particular, we have performed an image processing analysis to evaluate the kinematics and the dynamics of beating cardiac syncytia starting from the video registration of their contraction movement. We have found that the electromagnetic stimulation is able to counteract the *β*-adrenergic effect of isoproterenol and to elicit an antihypertrophic response.

## 1. Introduction

A core concept of tissue engineering is to understand the relationships between structures and functions in mammalian cells, tissues, and organs.

This knowledge is of fundamental importance during the growth and the development of tissue substitutes *in vitro*; in other words, the “morphogenesis” of tissue engineering constructs needs to be based not only on the use of molecules (e.g., growth factors) but also on the stimuli provided by the structural context (e.g., the natural/synthetic biomaterials with specific surface/volume properties, biocompatibility features, and mechanical properties) and provided by the biophysical context (e.g., the concentrated/distributed, perpendicular/tangential forces and stresses acting onto the plasma membrane, transmitted to the cytoskeleton and biochemically transduced; the deformations applied to the cell shape and transferred, via cytoskeleton, to the nuclear membrane and, as a consequence, to the DNA macromolecules in the form of heterochromatin and euchromatin; and the mechanical forces that influence, through cytoskeleton, the porosity of the nuclear envelop and, as a consequence, the trafficking of biochemical signals of mRNAs and microRNAs across the nuclear pores).

For example, a fluid shear stress [1–3] or ultrasounds [4] or biomaterial features [5] lead to the remodeling of bone matrix *in vitro*. In addition, the mechanical forces may also change the transcription more rapidly when they are transmitted directly into the nucleus via the cytoskeleton linked to nuclear envelop proteins [6].

The previous examples of structure/function relationship are comprehensible via the “tensegrity” theory [7–10]: during the *in vitro* morphogenesis inside bioreactors and biomaterials, the biophysical forces establish an equilibrium, the “tensegrity,” suitable to alter the transcription [11, 12].

Specifically, a modulation of the cell behavior is well proved by the cardiomyocytes subjected to the mechanical forces induced by an electromagnetic field [13, 14]. However, the effects of the electromagnetic fields are controversial. A work showed no main effects on heart function [15], whereas others suggested unfavorable consequences, such as arrhythmias and tachycardia [16, 17]. In addition, some studies showed that basal heart rate was either decreased and coupled with arrhythmias or increased with occurrence of tachycardia [18, 19].

In the heart, the *β*-adrenergic receptors (*β*ARs), associated to G proteins, play a crucial role in the regulation of the cardiac function [20, 21]; the stimulation of *β*_1_ARs and *β*_2_ARs increases the cardiac rate via cAMP production [20].

In this work, we have designed an *in vitro* model of murine ventricular cardiac tissue in order to study the contraction movement under electromagnetic and/or *β*-adrenergic stimulation, addressing, in particular, the inotropic and trophic effects.

## 2. Materials and Methods

### 2.1. Beating Mouse Cardiac Syncytia

Spontaneously beating cardiac syncytia were obtained from the hearts of 1- to 2-day-old CD-1® mouse pups (Charles River Laboratories Italia, Calco, Italy), as previously described [22–24] with some modifications. Briefly, beating primary cultures of murine cardiomyocytes were prepared *in vitro* as follows: the hearts were quickly excised, the atria were cut off, and the ventricles were minced and digested by incubation with 100 *μ*g/ml type II collagenase (Invitrogen, Carlsbad, CA) and with 900 *μ*g/ml pancreatin (Sigma-Aldrich, Milan, Italy) in ADS buffer (0.1 M HEPES, 0.1 M d-glucose, 0.5 M NaCl, 0.1 M KCl, 0.1 M NaH_2_PO_4_•H_2_O, 0.1 M MgSO_4_) for 15 min at 37°C. The resulting cell suspension was preplated for 2 h at 37°C to reduce the contribution of nonmyocardial cells. The unattached, cardiomyocyte-enriched cells remaining in suspension were collected, plated onto collagen-coated 35 mm Petri dishes, and covered by DMEM containing 10% horse serum, 5% fetal bovine serum, and 1× gentamicin (Roche Molecular Biochemicals, Indianapolis, IN). About 3 × 10^5^ cardiomyocytes were cultured in each Petri dish at 37°C and 5% CO_2_ to form a spontaneously beating cardiac syncytium (i.e., a cardiac cell culture made by multilayers of contracting cardiomyocytes as in our previous works [25, 26]).

### 2.2. Experimental Conditions

On day 3 of culture, at a constant temperature of 37°C and 5% CO_2_, each syncytium was observed via a movie capture system (ProgRes C5, Jenoptik, Germany) in four different conditions: untreated control (CTRL); stimulus via *β*-adrenergic isoproterenol (ISO, 10 *μ*M; Sigma-Aldrich, Milan, Italy); stimulus via an electromagnetic field (EMF; see below for details); and stimulus via both isoproterenol and electromagnetic field (ISO + EMF). In particular, for each condition, AVI videos (duration, 20 s) of 20 beating syncytia were collected every 3 min, permitting us to specifically study the average contraction pattern during the time interval 27–39 min.

### 2.3. Electromagnetic Bioreactor

The electromagnetic bioreactor used here has been previously investigated in terms of biological effects [27–31] and in terms of numerical dosimetry and physical parameters (induced electric field, induced electric current, and induced forces) [13]. The setup was based on two air-cored solenoids (see Figure 1 in [13]) connected in series, placed inside a cell incubator, and powered by a pulse generator (Biostim SPT from Igea, Carpi, Italy). The magnetic induction field (module, circa 3 mT; frequency, 75 Hz) was perpendicular to the seeded cells. In particular, in our experimental setup

**Figure 1:**
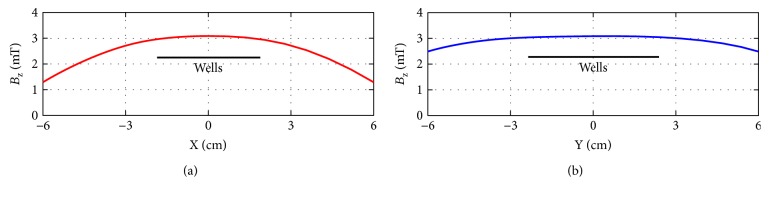
Magnetic induction field. Vertical component *B*_Z_ (in the Z direction) of the magnetic induction field B inside the electromagnetic bioreactor versus the X and the Y directions (panels (a) and (b), resp.). *B*_X_ and *B*_Y_ were negligible. The wells used for cell culture were in the region of field's quasihomogeneity (black horizontal lines in the center of the bioreactor).

- the electric current in the solenoids' wire ranged from 0 to 319 mA in 1.36 ms;
- in order to optimize the spatial homogeneity of the magnetic induction field, especially in the central region where the cells were stimulated, the two solenoids were supplied by the same electric current and their dimensions and distance were comparable; the spatial homogeneity was calculated in silico [13] and verified inside the cell incubator by means of a Hall effect gaussmeter (Figure 1());
- the maximum electromagnetic energy density applied to the cells was about 3.18 joule/m^3^ and, using a thermocouple, we observed no EMF-induced heating;
- during the same time interval of the electromagnetic stimulation, control cells were placed into another but identical incubator with no EMF.

### 2.4. Registration of the Syncytium Movement via the Apposition of Software Markers

By the Video Spot Tracker (VST) program, which is used to track the motion of one or more spots in an AVI video file (http://cismm.web.unc.edu/software/), in each video, we have systematically selected 30 spots or markers onto the first video frame, according to the same orthogonal grid [32, 33]. By starting the videos in VST, frame by frame, the program followed and registered the spatial-temporal coordinates *x*, *y*, and *t* for each marker, as previously described [25]. The coordinates *x* and *y* are expressed in pixel, whereas the coordinate *t* is in s.

### 2.5. Kinematics and Dynamics of the Beating Syncytium

By an algorithm based on the Matlab programming language (The MathWorks Inc., Natick, MA), frame by frame and for each marker, we have studied the kinematics and the dynamics of the beating cardiac syncytia, as previously described [25, 26, 34] (see Appendix below for the mathematical details). In particular, in this work, we have evaluated the syncytium contraction in terms of maximum contraction displacement [pixel], contractility (maximum contraction velocity) [pixel/s], and contraction acceleration [pixel/s^2^].

### 2.6. Immunofluorescence Analysis

Isolated cardiomyocytes were cultured in monolayer in a humidified atmosphere of 5% CO_2_ at 37°C for 48 h in the four preceding conditions. The cardiomyocytes were then fixed with 4% *w*/*v* paraformaldehyde (Sigma-Aldrich) in PBS (EuroClone, Pero, Italy) for 10 min at 4°C. The cells were washed with PBS and permeabilized with a solution of 0.2% *v*/*v* Triton X-100 (Sigma-Aldrich) in PBS for 10 min at 4°C and for further 30 min at room temperature.

The cells were blocked and incubated overnight with the murine monoclonal antibody MHC obtained from hybridoma (MF20, 1 : 5 *v*/*v*; Developmental Studies Hybridoma Bank, University of Iowa), which is able to recognize the sarcomeric myosin expressed by differentiated cardiomyocytes. Subsequently, the cells were incubated for 45 min at room temperature with a secondary antibody (anti-mouse Cy3, 1 : 50 *v*/*v*; Jackson ImmunoResearch, Newmarket, UK) conjugated to a fluorescent probe.

The cells were then observed with a Nikon Eclipse Ti microscope. The immunofluorescence was quantified by ImageJ software (https://imagej.nih.gov/ij/index.html).

### 2.7. Statistics

In order to compare the results between the different conditions, one-way analysis of variance (ANOVA) with post hoc least significant difference (LSD) test was applied, electing a significance level of 0.05. The results are expressed as mean ± 95% confidence interval for the differences between means.

## 3. Results

**Figure 2:**
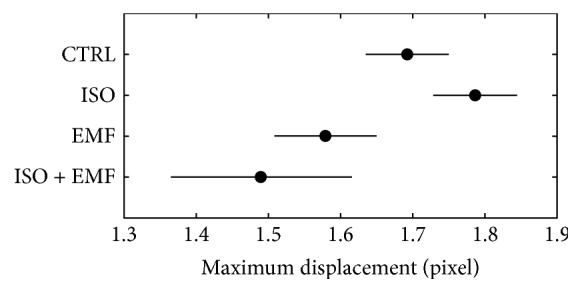
Mean maximum contraction displacement (during the time interval 27–39 min). In terms of kinematics, in comparison with control (CTRL), the isoproterenol (ISO) showed a nonsignificant positive inotropic effect (*p* > 0.05) and the electromagnetic stimulation (EMF) caused a nonsignificant negative inotropic action (*p* > 0.05). The simultaneous use of pharmacological and physical stimulation (ISO + EMF) significantly reduced the positive inotropic effect of ISO (*p* < 0.05), giving an overall significant negative inotropic action in comparison with CTRL (*p* < 0.05). The horizontal bars are the 95% confidence intervals for the differences between means according to LSD (least significant difference) statistical test: there is a statistically significant difference between the means with nonoverlapping bars (*n* = 20 syncytia for each condition).

**Figure 3:**
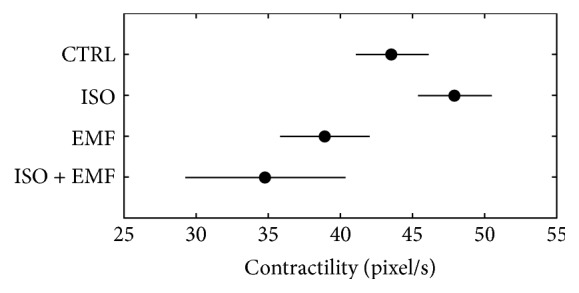
Mean contractility (during the time interval 27–39 min). In terms of kinematics, in comparison with control (CTRL), the isoproterenol (ISO) showed a nonsignificant positive inotropic effect (*p* > 0.05) and the electromagnetic stimulation (EMF) caused a nonsignificant negative inotropic action (*p* > 0.05). The simultaneous use of pharmacological and physical stimulation (ISO + EMF) significantly reduced the positive inotropic effect of ISO (*p* < 0.05), giving an overall significant negative inotropic action in comparison with CTRL (*p* < 0.05). The horizontal bars are the 95% confidence intervals for the differences between means according to LSD (least significant difference) statistical test: there is a statistically significant difference between the means with nonoverlapping bars (*n* = 20 syncytia for each condition).

In terms of kinematics (Figures 2 and 3), in comparison with the control, the isoproterenol showed a nonsignificant positive inotropic effect (*p* > 0.05) and the electromagnetic stimulation caused a nonsignificant negative inotropic action (*p* > 0.05). The pharmacological-physical stimulation significantly reduced the positive inotropic effect of isoproterenol (*p* < 0.05), giving an overall significant negative inotropic action in comparison with the control (*p* < 0.05).

**Figure 4:**
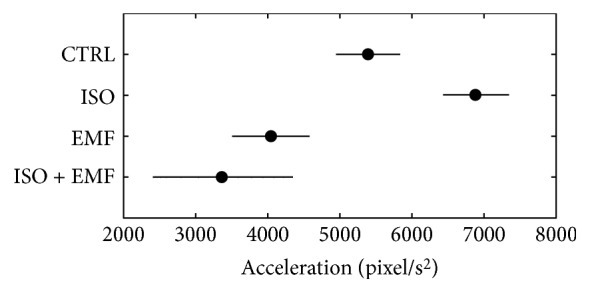
Mean contraction acceleration (during the time interval 27–39 min). In terms of dynamics, in comparison with control (CTRL), the isoproterenol (ISO) showed a significant positive inotropic effect (*p* < 0.05) and the electromagnetic stimulation (EMF) caused a significant negative inotropic action (*p* < 0.05). The simultaneous use of pharmacological and physical stimulation (ISO + EMF) significantly reduced the positive inotropic effect of ISO (*p* < 0.05), giving an overall significant negative inotropic action in comparison with CTRL (*p* < 0.05). The horizontal bars are the 95% confidence intervals for the differences between means according to LSD (least significant difference) statistical test: there is a statistically significant difference between the means with nonoverlapping bars (*n* = 20 syncytia for each condition).

In terms of dynamics (Figure 4), in comparison with the control, the isoproterenol showed a significant positive inotropic effect (*p* < 0.05) and the electromagnetic stimulation caused a significant negative inotropic action (*p* < 0.05). The pharmacological-physical stimulation significantly reduced the positive inotropic effect of isoproterenol (*p* < 0.05), giving an overall significant negative inotropic action in comparison with the control (*p* < 0.05).

**Figure 5:**
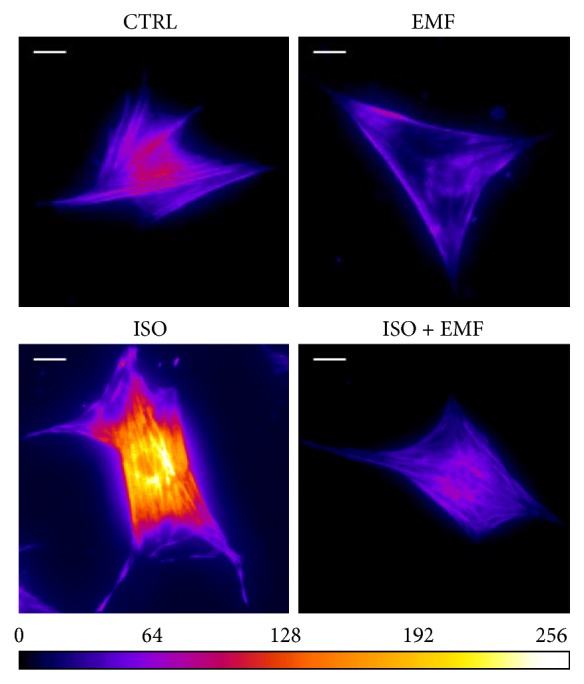
Immunofluorescence. A Fire LUT was applied using ImageJ in order to show the levels of MF20 immunofluorescence after 48 h of culture (white scale bar, 10 *μ*m; color scale in the range of 0–256 [arbitrary unit]). In comparison with control (CTRL), the isoproterenol (ISO) showed an enhancement of the fluorescence, whereas the electromagnetic stimulation (EMF) caused a reduction. The simultaneous use of pharmacological and physical stimulation (ISO + EMF) weakened the effect of ISO, giving an impairment in comparison with CTRL. The physically stimulated cultures showed an antisarcomeric effect of the electromagnetic field in the long term.

**Figure 6:**
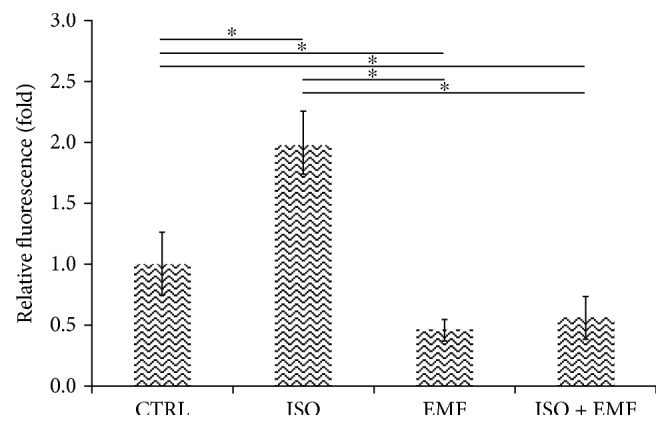
Quantitative immunofluorescence. After 48 h of culture, in comparison with control (CTRL), the isoproterenol (ISO) showed a significant prosarcomeric effect (^∗^*p* < 0.05) and the electromagnetic stimulation (EMF) caused a significant antisarcomeric action (^∗^*p* < 0.05). The simultaneous use of pharmacological and physical stimulation (ISO + EMF) significantly reduced the effect of ISO (^∗^*p* < 0.05), giving an overall significant antisarcomeric action in comparison with CTRL (^∗^*p* < 0.05). The normalized data are expressed as mean fold ± 95% confidence interval (*n* = 20 cells for each condition).

In addition, in isolated cardiomyocytes after 48 h of culture (Figures 5 and 6), in comparison with the control, the isoproterenol showed a significant prosarcomeric effect (*p* < 0.05) and the electromagnetic stimulation caused a significant antisarcomeric action (*p* < 0.05). The simultaneous use of pharmacological and physical stimulation significantly reduced the effect of isoproterenol (*p* < 0.05), giving an overall significant antisarcomeric action in comparison with the control (*p* < 0.05).

## 4. Discussion

The mouse is in the center of the research due to the high potential in manipulating its genome and the consequent availability of models of cardiovascular diseases. Using *in vitro* beating primary murine ventricular cardiomyocytes, we have studied the alteration of their contraction following the mechanical forces induced by an electromagnetic field and/or a *β*-adrenergic stimulation (10 *μ*M isoproterenol) [13, 14].

Studies about the action of electromagnetic fields on the heart function are of interest due to the high rate of cardiac diseases and the everyday environmental electromagnetic exposure [35]. However, the epidemiological studies have been indecisive [18, 36].

By means of an electromagnetic bioreactor, previously described [27–29, 37–45], our preceding study showed that an exposure to a low-frequency EMF decreases the beat frequency of neonatal murine cardiomyocytes, frequency and amplitude of the intracellular calcium transients, the contraction force, the kinetic energy, and also the effects of the *β*-adrenergic stimulation [14].

In the present study, we have showed that a low-frequency electromagnetic stimulus was able to counteract both the basal inotropism and the *β*-adrenergically enhanced inotropism, probably due to the internalization of *β*_2_ARs [14] and/or the inhibition of T-type calcium channels via AA/LTE4 signaling pathway [46].

In addition, the anti-*β*-adrenergic response after short exposure (27–39 min) to EMF preempted an antisarcomeric/antihypertrophic effect due to a longer exposure (48 h); in other words, a prolonged underuse of the sarcomeric apparatus caused a down remodeling of it.

## 5. Conclusion

Although some epidemiological studies raise concerns about the low-frequency electromagnetic exposure [18, 36], this work suggests a potential application of that biophysical stimulus in the treatment of arrhythmias and hypertrophy. In particular, a weakening of the *β*-adrenergic sensibility can be significant in the ischemia-reperfusion injuries, where an abnormal depolarization could arise outside the normal conduction tissue causing life-threatening arrhythmias.

## Appendix

Being both contraction and relaxation active phases of the syncytium movement, we have defined *E* as the mean kinetic energy of a beating syncytium in a discrete video:

(A.1) $$\begin{array}{c} E=\frac{1}{2}A\frac{B}{NM}\overset{N}{\underset{i=1}{\sum }}\overset{M}{\underset{j=1}{\sum }}{\left|{\underset{¯}{v}}_{i,\;j}\right|}^{2}\;in\;joule, \end{array}$$

where ${\underset{¯}{v}}_{i,\;j}$ is the velocity of the marker *i* in the frame *j*, *M* is the total number of video frames, *N* is the total number of markers (*N* = 30), *A* is the constant related to the tissue mass, and *B* is the constant derived from the linear relation between the units meter and pixel in a bitmap AVI video at a given magnification. In (A.1), for each syncytium, in order to compare the four different experimental conditions [untreated control (CTRL), stimulus via *β*-adrenergic isoproterenol (ISO), stimulus via an electromagnetic field (EMF), and stimulus via both isoproterenol and electromagnetic field (ISO + EMF)], there was no need to know the mass of the beating tissue or the *A* constant, because that mass and constant were the same in the four different conditions and the spot markers were juxtaposed in the same grid positions. In addition, there was no need to know the video metrics or the *B* constant, because that metrics and constant and the video magnification were the same at all conditions.

According to Sonnenblick et al. [47, 48], the maximum contraction velocity is an indicator of contractility. As a consequence, in order to study a possible inotropic effect under a kinematic point of view, for each marker during its beating, we have identified both the maximum contraction velocity and the maximum contraction displacement; then, we have calculated the mean contractility [pixel/s] (Figure 3) and the mean maximum contraction displacement [pixel] (Figure 2), respectively.

In order to study a possible inotropic effect under a dynamic point of view, we have evaluated the syncytium contraction by the Hamiltonian mechanics. The so-called Hamiltonian function *H* is the sum of the kinetic and potential energy. Assuming that, during the whole video observation, there was a plentiful source of available glucose from the culture medium and that the subsequent ATP production and distribution were isotropic, *P*_ATP_, the ATP-related potential energy for the contraction movement, could be supposed constant in time and in space. As a consequence, the Hamilton differential equations to describe the syncytium movement were

(A.2) $$\begin{array}{c} F_{x}=-\frac{\partial H}{\partial x}=-\frac{\partial }{\partial x}\left(E_{ATP}+P_{ATP}\right)=-\frac{\partial E_{ATP}}{\partial x}\;in\;newton,\\ F_{y}=-\frac{\partial H}{\partial y}=-\frac{\partial }{\partial y}\left(E_{ATP}+P_{ATP}\right)=-\frac{\partial E_{ATP}}{\partial y}\;in\;newton, \end{array}$$

where *F*_*x*_ and *F*_*y*_ are the orthogonal components of the contraction force $\underset{¯}{F}$ and *E*_ATP_ = *E*_ATP_(*x*, *y*, *t*) is the kinetic energy function of the beating syncytium.

Then, we have defined *F*_mean_ as the normalized mean contraction force, that is, as the mean contraction acceleration (Figure 4()) of a beating syncytium in a discrete video:

(A.3) $$\begin{array}{c} F_{mean}=\frac{1}{AB}\frac{1}{NM}\overset{N}{\underset{i=1}{\sum }}\overset{M}{\underset{j=1}{\sum }}\left|{\underset{¯}{F}}_{i,\;j}\right|\;in\;\frac{pixel}{s^{2}}. \end{array}$$
